# Nontuberculous mycobacterial skin and soft tissue infection in Hawaiʻi

**DOI:** 10.1186/s12879-022-07345-y

**Published:** 2022-04-11

**Authors:** Darcy S. Tokunaga, Andrea M. Siu, Sian Yik Lim

**Affiliations:** 1 Hawaiʻi Pacific Health, Honolulu, USA; 2 Hawaiʻi Pacific Health Research Institute, Honolulu, HI USA; 3 Hawaiʻi Pacific Health Medical Group, 98-1079 Moanalua Road, Suite 300, Aiea, Honolulu, HI 96701 USA; 4 Department of Family Medicine, University of Hawaiʻi at Manoa, John A. Burns School of Medicine, Honolulu, HI USA

**Keywords:** Nontuberculous mycobacterium, Skin infections, Soft tissue infections, Hawaiʻi

## Abstract

**Background:**

Hawaiʻi has the highest nontuberculous mycobacterial (NTM) lung infection prevalence in the United States. Limited data regarding skin and soft tissue infections (SSTI) due to NTM in Hawaiʻi exists. This study describes patient demographics, clinical courses of infection, treatment patterns, and clinical outcomes of NTM SSTIs in Hawaiʻi.

**Methods:**

A retrospective chart review (n = 50) of patients diagnosed and treated at Hawaiʻi Pacific Health facilities for NTM SSTIs between January 2010 and July 2021 was conducted. Patient demographics, clinical course, and treatment data were collected from electronic medical records.

**Results:**

Half of the patient population consisted of females, and the average age of patients during infection was 49 years (SD = 25.6). The majority of cases (80%) were caused by rapidly growing mycobacteria (RGM), most commonly *Mycobacterium abscessus*. NTM SSTI by race were Asian (48%), White (28%), and Native Hawaiian and Other Pacific Islanders (16%). Almost all Asian patients with NTM SSTI were Filipino or Japanese. Diagnosis was frequently delayed. The average time to diagnosis was 116 days. Most patients achieved complete resolution (72%) following a prolonged course of antimicrobial treatment (mean = 196 days) with surgical debridement.

**Conclusion:**

Increased awareness among physicians and the community of non-mycobacterial skin infections is essential in Hawaiʻi due to the high prevalence of NTM and the high percentage of predisposed populations. Increased awareness of NTM could reduce delayed diagnosis and improve patient care. Further studies are required to inform optimal treatment and diagnostic strategies, improve patient outcomes, and aid public health surveillance efforts.

## Introduction

Nontuberculous mycobacteria (NTM) are environmental pathogens that are commonly found in water and soil [1, 2]. NTM refers to mycobacteria other than Mycobacterium tuberculosis complex and *M. leprae*. NTM can be further classified by growth rate and pigment formation, with rapidly growing mycobacteria growing in less than 7 days. The most common rapidly growing mycobacteria (RGM) include *M. abscessus*, *M. chelonae*, and *M. fortuitum*. Slow-growing NTM and RGM are differentiated based on their coloration and include *M. marinum*, *M. avium* complex (MAC), and *M. kansaii* [1].

NTM are increasingly recognized as a source of infection [2]. Clinical manifestations of NTM infection are predominantly pulmonary but include lymphadenitis, skin infections, musculoskeletal disease, and disseminated disease [3]. Non-pulmonary NTM skin and soft tissue infection (SSTI) occur secondary to direct inoculation and contamination during events such as penetrating injuries, trauma, injections, and surgical procedures [4, 5]. Due to the rarity and indolent nature of non-pulmonary NTM SSTIs and deficiency in diagnostic tools, diagnosis is challenging and often delayed. Additionally, population-level data on non-pulmonary SSTIs of NTM are limited [6].

Hawaiʻi has the highest prevalence of NTM lung infection in the United States, reporting NTM infection rates nearly four times the national average [7]. Additionally, Asian Americans and Pacific Islanders are at increased risk of NTM lung infections independent of the geographic area of residence [6]. Most residents in Hawaiʻi are of Asian, Native Hawaiʻi, or Pacific Islander descent. Limited literature exists on NTM pulmonary infections in Hawaiʻi, which is characterized by a high-risk setting with predisposed populations (Asian American/Pacific Islanders) [8]. Even less information about NTM non-pulmonary SSTIs is available, making diagnosis and treatment of this rare condition especially challenging in a diverse environment such as Hawaiʻi. This study describes the clinical characteristics, demographics, clinical course, treatment patterns, and clinical outcomes of non-pulmonary NTM SSTIs for patients treated over 10 years in an extensive health care system in Hawaiʻi.

## Methods

### Research setting and design

A retrospective chart review was conducted of patients diagnosed and treated for NTM SSTI at four medical centers within the Hawaiʻi Pacific Health (HPH) system from 2010 to 2021. Hawaiʻi Pacific Health is Hawaiʻi’s largest health care provider with four medical centers and over 70 clinics across the state [9]. The study was reviewed and determined to be exempt from Institutional Review Board approval by the HPH Research Institute. The study was conducted in accordance with all applicable regulations, including the United States Common Rule and the Declaration of Helsinki.

### Data collection

Patients with positive culture for nontuberculous mycobacteria at HPH from January 1, 2010, to July 1, 2021, were identified, and diagnosis of NTM SSTI was confirmed through a manual medical chart review (n = 50). Mycobacterium speciation and susceptibility data were extracted from positive cultures analyzed by certified clinical laboratories. Data collected included demographics such as sex, race, co-morbidities, and age. Race data were based on self-reports of the patient’s primary race [10, 11]. The race/ethnicity variable is created from categories consistently available across all hospitals in Hawai‘i, as per Hawai**ʻ**i Health Information Corporation, which collects health care data for the state of Hawai**ʻ**i. Race/ethnicity data are provided by patient self-report at intake and includes one primary race. Mixed-race individuals are represented by self-report of their primary race identification. Manual chart review yielded details of the infection’s clinical course, such as the type of soft tissue infection, the site of infection, and the diagnostic procedure utilized. Time to diagnosis and duration of treatment and infection were manually calculated based on medical chart review. The time to diagnosis was defined as the number of days from symptom onset first recorded by the medical provider to the first recorded NTM positive culture result. Infection duration was measured as the time from the first onset of symptoms to discontinuation of antibiotic treatment due to symptom resolution.

Previous surgeries included procedures requiring local, regional, or general anesthesia and injections. Prior physical traumas included blunt force or penetrating injuries to the area where the infection occurred. Additionally, immunosuppressive medications taken by each patient were recorded as an additional predisposing factor to infection. Patients were considered immunocompromised if they received chemotherapy or immunosuppressive medication or were diagnosed with rheumatic disease, human immunodeficiency virus (HIV), or acquired immunodeficiency syndrome (AIDS). The treatment course was described for each patient by recording the treatment received and the duration of the infection. The outcome of each case of infection, including complete recovery, mortality, recurrent infections, and dissemination, was also recorded.

### Statistical analysis

Descriptive statistics were utilized to summarize patient demographics, infection characteristics, treatment methods, and outcomes. The mean and standard deviation was calculated for continuous variables, and frequency and the percentage were calculated for categorical variables. Analyses were conducted using Stata v15.1 (StataCorp, College Station, TX).

## Results

### Clinical characteristics of non-tuberculous mycobacterium skin and soft tissue infections

NTM SSTI isolates were recorded for the 50 patients (25 female, 25 male) with a positive culture and/or polymerase chain reaction (PCR) and were diagnosed as NTM SSTI from 2010 to 2021. The clinical characteristics of patients are summarized in Table 1. The average age of patients was 48.5 years. NTM SSTIs were seen most in Asians (48%), followed by Whites (28%) and Native Hawaiian and Other Pacific Islanders (16%). Of the Asian patients, 15 were Filipino (63%), 7 were Japanese (29.1%), and 2 were classified as other Asian.

**Table 1:** Clinical characteristics of patients with NTM skin and soft tissue infections

Demographic variables (n = 50)	Mean ± SD/Freq (%)
Infection age (years)	48.50 ± 25.60
Sex	
Female	25 (50)
Male	25 (50)
Race	
White	14 (28)
Asian	24 (48)
NHOPI	8 (16)
Black	2 (4.0)
Other	2 (4.0)
Underlying comorbidities	
Diabetes mellitus	10 (20.0)
Malignant neoplasm	10 (20.0)
Chronic hepatic disease	1 (2.0)
Rheumatic disease	6 (12.0)
HIV/AIDS	2 (4.0)
Chronic kidney disease	7 (14.0)
Immunosuppresive treatment	2 (4.0)
Corticosteroid treatment	11 (22.0)
COPD	2 (4.0)
Dialysis	6 (12.0)
Organ transplant	1 (1.4)
Prior chemotherapy	6 (8.6)
Medical/surgical procedure	23 (45)
Prior skin trauma	15 (50)
Site of infection	
Upper extremity	14 (28.0)
Lower extremity	8 (16.0)
Head or neck	12 (24.0)
Trunk	15 (30.0)
Others	1 (2.0)
Time to diagnosis (days)	115.96 ± 263.14
Interval between illness onset and diagnosis	
< 7 days	2 (4.0)
7 days–1 month	17 (34.0)
1–2 months	13 (26.0)
2–3 months	4 (8.0)
> 3 months	14 (28.0)

**Fig. 1:**
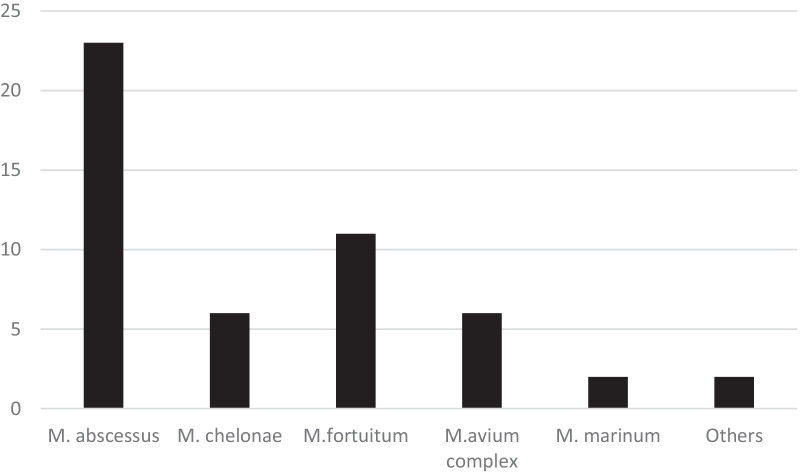
Mycobacterial species causing skin and soft tissue infection

The most common species identified were RGM, consisting of *M. abscessus* (23 cases), *M. fortuitum* (11 cases), and *M. chelonae* (6 cases). Other skin infections were caused by MAC, *M. marinum*, and 2 other cases from other species (Fig. 1). The average patient age at infection for *M. abscessus* and *M. chelonae* were 60.65 years and 49.17 years, respectively. The average patient age for *M. fortuitum* was 34 years (Table 2). Cellulitis/Nodules/Abscess were the most common presentation. The upper and lower extremities were the most commonly involved (44%) body areas. Most cases were localized infections. Disseminated NTM infection occurred in one patient caused by MAC.

**Table 2:** Demographic variables by mycobacterial species

Demographic variable	*M. abscessus* (n = 23), Mean ± SD/Freq (%)	*M. chelonae* (n = 6), Mean ± SD/Freq (%)	*M. fortuitum* (n = 12), Mean ± SD/Freq (%)	*M. avium* complex (n = 6), Mean ± SD/Freq (%)	*M. marinum* (n = 2), Mean ± SD/Freq (%)	Others (n = 1), Mean ± SD/Freq (%)
Sex						
Male	9 (39)	3 (50)	7 (64)	3 (50)	2 (100)	1 (50)
Female	14 (61)	3 (50)	4 (36)	3 (50)	0 (0)	1 (50)
Infection age (years)	60.65 ± 18.40	49.17 ± 20.80	34.00 ± 28.10	41.33 ± 33.00	42.50 ± 20.50	14.00 ± 17.00
Site of infection						
Upper extremity	5 (22)	2 (33)	2 (18)	3 (50)	2 (100)	0 (0)
Lower extremity	3 (13)	1 (17)	3 (27)	0 (0)	0 (0)	1 (50)
Head or neck	6 (26)	0 (0)	3 (27)	3 (50)	0 (0)	0 (0)
Trunk	9 (39)	2 (33)	3 (27)	0 (0)	0 (0)	1 (50)
Others	0	1 (17)	0 (0)	0 (0)	0 (0)	0 (0)
Time from symptom onset to dx (days)	59.7 ± 58.0	57.33 ± 42.5	85.91 ± 168.1	112.5 ± 111.6	1164.5 ± 806.8	66.5 ± 82.7

### Risk factors for non-tuberculous mycobacterium skin and soft tissue infections

The most common comorbidities (Table 1) were diabetes mellitus (20%), a history of malignant neoplasm (20%), and rheumatic disease (12%). Other risk factors for infection included concurrent corticosteroid treatment (22%), prior chemotherapy (8.6%), and other immunosuppressive medications (4%). Immunosuppression was found in 26 of the 50 patients (52%).

Twenty-three patients (45.1%) had prior surgical/medical procedures at the site of infection. Surgical procedures included breast implantation, dermatologic surgery (lipoma resection, skin biopsy), orthopedic surgery (open reduction and internal fixation for wrist fracture), peritoneal dialysis catheter placement, and intravenous catheter placement. In patients with NTM SSTI after surgical or medical procedures, 95% of the cases were due to RGM.

Fifteen patients (30%) had a history of skin trauma/skin injury at the site of infection. These included cuts and scratches sustained during various activities, including wood-splinter injuries aquatic-related injuries (injuries sustained while fishing, shark bites). Three patients had skin injury related to non-surgical cosmetic and body modifications related to recent tattooing (1 case of *M. abscessus*, 2 cases of *M. chelonae*). The 2 patients with *M. marinum* SSTI infections reported exposure to seawater in addition to skin trauma. In Table 3, we present information of patients who had iatrogenic trauma (patients who had prior surgical/medical procedures at site of infection, and recent tattooing) as compared to patients who had accidental trauma and patients who had no trauma history as reported in medical records. Most cases who had iatrogenic trauma were due to Rapidly Growing Mycobacteria, while those who had accidental trauma had a more heterogenous cause of infection which included RGM, *M Avium*, and *M Marinum*.

**Table 3:** Demographic variables of patients with iatrogenic trauma, accidental trauma and no exposure determined

Demographic variable	Iatrogenic trauma	Accidental trauma	No trauma
	(n = 26)	(n = 12)	(n = 12)
Sex			
Male	9 (34.6)	11 (91.7)	5 (41.7)
Female	17 (65.4)	1 (8.3)	7 (58.3)
Infection age (years)	52.4 ± 19.2	54.4 ± 24.5	34.2 ± 34.4
Mycobacterium species			
* M. Abscessus*	16 (61.5)	3 (25.0)	4 (33.3)
* M. Chelonae*	4 (15.4)	1 (8.3)	1 (8.3)
* M. Fortuitum*	5 (19.2)	4 (33.3)	3 (25.0)
* M. Avium*	0	2 (16.7)	4 (33.3)
* M. Marinum*	0	2 (16.7)	0
Other	1 (4.0)	0	0
Site of infection			
Upper extremity	5 (19.2)	7 (5.8)	2 (16.7)
Lower extremity	2 (7.7)	3 (25.0)	3 (25.0)
Head or neck	5 (19.2)	1 (8.3)	6 (50)
Trunk	14 (53.8)	0 (0)	1 (8.3)
Others	0 (0)	1 (8.3)	0 (0)
Time from symptom onset to dx (days)	81.9 ± 116.8	252.1 ± 496.9	58.1 ± 16.8

### Diagnosis of non-tuberculous mycobacterium skin and soft tissue infections

Approximately 50% of patients were diagnosed 1 month after the onset of symptoms. The mean time from symptoms onset to diagnosis was 116 days (SD ± 263.1 days). MAC and *M. marinum* infections had a longer time to diagnosis at 112.5 days and 1164.5 days, respectively. Initial acid-fast bacillus testing was only positive in 26% of patients.

### Treatment and outcome of non-tuberculous mycobacterium skin and soft tissue infections

Thirty-two percent of patients were treated with antibiotic therapy alone, and 62% received surgical debridement and antibiotic treatment. The average length of treatment with antibiotics was 196 days (SD ± 429 days). The most common antibiotics used were clarithromycin (32%), other macrolides (24%), fluoroquinolones (40%), and trimethoprim-sulfamethoxazole (18%). Table 4 summarizes the treatment and treatment outcomes by species in our NTM SSTI cohort.

**Table 4:** Treatment and response by mycobacterial species

	*M. abscessus *(n = 23), Mean ± SD/Freq (%)	*M. chelonae* (n = 6), Mean ± SD/Freq (%)	*M. fortuitum* (n = 12), Mean ± SD/Freq (%)	*M. avium* complex (n = 6), Mean ± SD/Freq (%)	*M. marinum* (n = 2), Mean ± SD/Freq (%)	Others (n = 1), Mean ± SD/Freq (%)
Time on antibiotics	129.9 ± 167.2	540.2 ± 1160.1	168.9 ± 209.1	94.0 ± 79.6	134.0 ± 99.0	716
Mortality	1 (4)	1 (16)	0	0	0	0
Cure rate	19 (83)	3 (50)	10 (91)	2 (33)	1 (50)	1 (100)
Treatment						
Antibiotics alone	6 (26)	3 (50)	3 (25)	1 (16.7)	2 (100)	1 (100)
Surgical intervention only	0	1 (16.7)	0	1 (16.7)	0	0
Antibiotics and surgery	16 (69.6)	2 (33.3)	9 (75)	4 (66.7)	0	0
Other	1 (4)	0	0	0	0	1 (100)

Most cases of NTM SSTI were resolved, with 72% of patients having complete recovery. There were 4 cases in which the infection was recurrent. Two cases of mortality were noted, with both patients being immunosuppressed.

### Trends of non-tuberculous mycobacterium skin infections

**Fig. 2:**
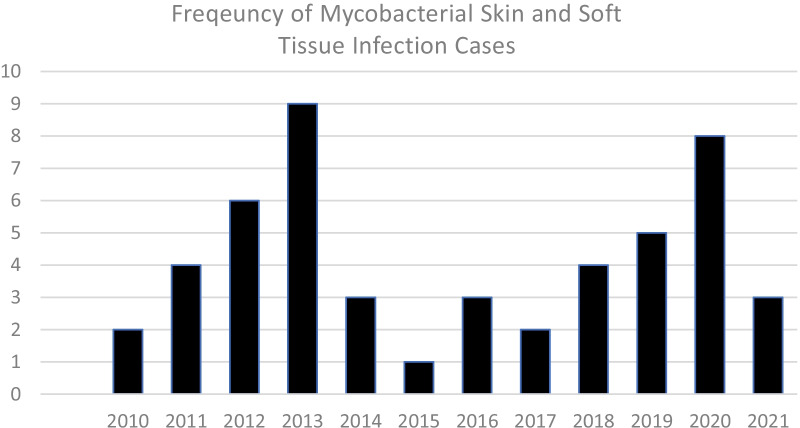
Frequency of mycobacterial skin and soft tissue infection cases 2009–2021

From 2010 to 2021, 50 patients with culture-proven non-tuberculous mycobacterium were identified. The number of cases each year was variable, an average of 3.85 cases per year (range 1–9) with no visible trend noted (Fig. 2).

## Discussion

NTM encompasses more than 150 different mycobacteria species and is increasingly a cause of human pathology [2]. NTM most commonly causes pulmonary infections. NTM SSTIs are relatively rare, frequently underrecognized, and diagnostically challenging. This study describes NTM mycobacterial skin infections within a large health care system in Hawaiʻi [5]. No prior studies have reported NTM skin infections in Hawaiʻi. Defining the epidemiology in the environment, animals, and healthcare is helpful in disease prevention, public health surveillance efforts, and policy and organizational planning [12].

In contrast to NTM pulmonary disease, which primarily occurs in females [8], NTM SSTI affected equal proportions of males and females in our case series. Patients with NTM SSTI had an average age of 48.5 years, which is younger than the average age of NTM pulmonary disease patients previously reported in Hawaiʻi (Mean age 66, SD 16 years) [13]. For NTM pulmonary disease, the most common NTM species identified were MAC 64%, *M. fortuitum* 24%, and *M. abscessus* 19% [8]. For SSTIs in our study, fewer patients had infections due to MAC, with most infections caused by RGM (80% of patients).

Higher rates for NTM pulmonary infections, primarily due to *M. avium*, have been reported in East Asian populations [14]. For NTM pulmonary infections in persons living in Hawaiʻi, Asians were at the most significant risk while Native Hawaiian and Pacific Islanders and Whites were at lower risk [8]. In our study, approximately 50% of NTM SSTI were seen in Asians, while Whites and Native Hawaiians and Pacific Islanders consisted of roughly 28% and 16% of cases, respectively. Interestingly, almost all SSTI in Asian patients were seen in Filipino and Japanese patients, although Filipino and Japanese only make up about 29% of the population in counties that our health care system primarily serves [15]. The reasons for this are unclear. However, assessing the role of race and ethnicity in NTM SSTI is complex because racial categorizations likely reflect a complex interaction of behavioral, cultural, biological, and genetic factors [8].

Exposure to contaminated mediums increases the risk of NTM SSTI. In humans, infection is almost always contracted from the environment, although the source of infection may not be identified [1]. NTM are widespread in the environment and have been isolated from various environmental sources, including water, soil, and domestic and wild animals [16]. Increased prevalence of NTM in Hawaiʻi may be due to environmental conditions, including having soil with high humic acid, a component associated with increased rates of mycobacteria [17–19]. Because soil is a source of waterborne pathogens, water contamination may occur, leading to an increased risk of exposure to water and soil sources in Hawaiʻi [8].

In our case series, almost all cases with a history of prior surgical procedures were due to RGM. RGM is widely distributed in nature and isolated from soil, dust, water, and hospital environments (including hospital tap water)[4]. In particular, *M. fortuitum* and *M. abscesses* are relatively resistant to standard disinfectants such as chlorine, alcohols, and formaldehyde and are a common cause of nosocomial infections, including dialysis-associated infections, long term central intravenous catheters, injections, and plastic surgery procedures [20–23]. Therefore, preventative steps should be taken in medical settings, such as avoiding contact with catheters, medical instruments with tap water, and refraining from washing open wounds with tap water [1, 5].

Immunosuppression is a risk factor for NTM infections, particularly in patients with HIV, patients with organ transplants [24], cancer patients [25], or biologic treatment, especially patients using anti-TNF therapies [26]. However, NTM SSTIs can occur in patients who are not immunocompromised, especially when the physical protection from the skin is breached by penetrating trauma. The skin serves as a physical barrier that protects our bodies from infection [16]. Penetrating skin trauma due to medical procedures/surgery (45% of cases in the current study) or skin injuries (30% of patients in the present study) is a major risk factor for NTM SSTIs. Skin injuries include lacerations sustained by contact with inanimate objects, puncture wounds from fish hooks/fish spines and bites from aquatic animals [27]. Individuals with preexisting wounds are susceptible to NTM SSTI after exposure.

The increasing popularity of cosmetic and body modifying procedures has been associated with an increase in NTM SSTIs [12]. This includes NTM SSTI related to tattooing [28]; there were 3 tattoo-related cases in our cases series. NTM SSTIs related to tattooing are primarily due to *M. chelonae*, although cases due to *M. abscesses* and other species have been reported [12, 29]. Most cases in the United States were associated with premixed black or grey ink due to contamination of nationally distributed ink products during the manufacturing process or dilution of black ink locally with tap water [12, 29]. More stringent regulations regarding standardization of tattoo ink preparation and use may be needed to ensure the safe conduct of cosmetic and body modifying procedures [12, 30].

The diagnosis of NTM SSTI is frequently challenging. There was a significant delay from the time of symptoms onset to diagnosis. The average time from symptom onset to diagnosis was 116 days. Approximately 50% of patients were diagnosed 1 month after onset of symptoms. A high degree of clinical suspicion is needed for the diagnosis and must emphasize assessing clinical context, morphological picture, and microbiology [1]. Identification of risk factors such as recent surgery, skin trauma or immunosuppression, or exposure may be helpful. NTM SSTI infection should be considered in any patient with a history of negative bacterial cultures or failed standard antibiotic therapy, [20]. Because the incubation period can be prolonged for more than 30 days, patients with atypical skin infections should be questioned about high-risk exposures to *M. marinum* up to 9 months after the onset of symptoms [31]. Other factors contributing to the delay in diagnosis include failure to do relevant cultures for specific mycobacteria [32], lack of communication between the clinician and the microbiology laboratory [12], lack of familiarity of the physician to specific mycobacterium diseases, and incorrect specimen collection [32].

There remains an unmet need for faster methods of diagnosis. Only 26% of patients had a positive acid-fast bacillus. Culture remains the gold standard for diagnosis, providing invaluable information about species-level identification and sensitivity testing to inform antibiotic selection. However, culture techniques are laborious and time-consuming [12]. PCR restriction enzyme analysis or DNA gene sequencing provides faster species-level identification and is increasingly utilized in clinical settings [33]. In recent years, matrix-assisted laser desorption ionization-time of flight mass spectrometry (MALDI-TOF) has increasingly been adopted for bacterial identification [12]. Current guidelines recommend that specimens be obtained by needle aspirations or surgical procedures. Care is needed to avoid sources of contamination especially tap water. Communication between clinicians and the microbiology laboratory is critical because clinical input is required to guide phenotypic and genotypic identification of non-tuberculous mycobacteria [1].

Standard management of NTM SSTI involves antibiotic treatment for 3–6 months [20]. In more complicated cases, surgical treatment (abscess drainage, removal of foreign bodies, extensive disease) is an adjunct to therapy. Specific species of mycobacterium, susceptibility profile, and patient immunological status are essential factors in determining treatment [34]. A multidisciplinary approach involving dermatology, infectious disease, histopathologists, and microbiologists is critical in successful diagnosis and management. No randomized control trials are available to guide treatment. Therefore, treatment is generally recommended by case reports, case series, susceptibility testing, and expert opinion [20].

For SSTIs due to RGM, MAC, and another mycobacterium, treatment is guided by cultures and sensitivity [35]. Therapy with at least two antibiotics, which the isolate is susceptible to for at least 4 months, is recommended [8]. Typical antibiotics used to treat infection include ciprofloxacin, levofloxacin, Trimethoprim-sulfamethoxazole (TMP-SMX), doxycycline, or clarithromycin [20]. Initially, antibiotics known to be active against mycobacteria are started, then changed based on culture’s sensitivity results [1]. For *M. marinum* with minimal disease, monotherapy with clarithromycin, doxycycline, minocycline, and TMP-SMX may be adequate treatment options in superficial infections. In contrast to other NTM, routine antimicrobial susceptibility testing is not required unless treatment failures are observed [17]. Most cases of non-mycobacterial SSTI resolve with treatment. However, recurrence occurred in approximately 10% of cases in our study.

Our study has several limitations. First, due to the retrospective nature of our research, the information in our study is limited to what could be obtained from medical records. There is a possibility that data within the medical records contained errors and inconsistencies. There was vague documentation of the timeline in the patient’s medical chart in a few cases. We did our best to approximate the duration from onset of symptoms to diagnosis and treatment duration. Secondly, our patient cohort was from a population based in Hawaiʻi. The results may not be generalizable to other healthcare settings. Furthermore, the number of cases in our case series is small due to the rarity of NTM SSTIs. Nevertheless, our data provide insight into NTM SSTI in an area with a high prevalence of non-tuberculous mycobacterium, with a population with a racial composition different from the contiguous United States.

## Conclusion

This study gives insight into the presence of NTM SSTI over a 10-year period in Hawaiʻi. Increased awareness among physicians and the community at large of non-mycobacterial skin infections is essential in Hawaiʻi due to the high prevalence of nontuberculous mycobacteria and the higher percentage of predisposed populations. The diagnosis is frequently delayed, and a high degree of clinical suspicion is needed for the diagnosis. Identifying risk factors, including immunosuppression, prior exposure to contaminated mediums, and previous surgical procedures, are essential. Further studies are required to inform optimal treatment and diagnostic strategies, improve patient outcomes, and aid public health surveillance efforts.

## Data Availability

The datasets generated and/or analyses during the current study are not publicly available due to privacy concerns and the relatively small number of patient records included in the study but are available from the corresponding author on reasonable request. To request data from this study please email the corresponding author at limsianyik@gmail.com.

## Abbreviations

NTM: Nontuberculous mycobacteria
SSTI: Skin and soft tissue infection
HPH: Hawaiʻi Pacific Health
HIV: Human immunodeficiency virus
AIDS: Acquired immunodeficiency syndrome
PCR: Polymerase chain reaction
RGM: Rapidly growing mycobacterium
MAC: *M. avium* Complex
MALDI-TOF: Matrix-assisted laser desorption ionization-time of flight mass spectroscopy
TMP- SMX: Trimethoprim-sulfamethoxazole
